# Use of Biologic Agents in Ocular Manifestations of Rheumatic Disease

**DOI:** 10.1155/2012/203819

**Published:** 2011-12-15

**Authors:** Courtney L. Kraus, Susan M. Culican

**Affiliations:** Department of Ophthalmology and Visual Sciences, Washington University School of Medicine, St. Louis, MO 63110, USA

## Abstract

Biologic agents have dramatically shifted the treatment paradigm for rheumatic disease. Use of these agents can decrease disease burden, allow the patient to be weaned from corticosteroids, and reduce the likelihood of relapse. Eye disease associated with rheumatic conditions may present with a wide range of signs and symptoms. This coexisting pathology should not be overlooked and should be considered a reason for initiation or continuation of biologic therapy. Additionally, many of the ocular manifestations of rheumatic disease respond preferentially to specific targeting molecules. This paper summarizes the available studies on the use, efficacy, and safety of biologic agents in the treatment of ocular manifestations of rheumatic disease.

## 1. Introduction

Eye disease associated with rheumatic conditions may present with a wide range of signs and symptoms. The treating physician must be careful not to overlook ocular manifestations, as they can be quite subtle. Dry eye syndrome, acute and chronic anterior uveitis, panuveitis or vitritis, scleritis, keratitis, retinal vasculitis, and ischemic optic neuropathy represent ocular disorders frequently associated with rheumatic diseases. In patients presenting with nonspecific signs and symptoms, ocular findings may be the only clue to the final diagnosis. Alternatively, in patients with long-standing rheumatic disease, ophthalmic flares may suggest further deterioration or relapse.

The mainstay of management of inflammatory ocular conditions has been topical agents with occasional oral corticosteroid use. However, for some conditions these therapies are often inadequate. Biologic therapies (Table 1) have demonstrated efficacy in the control of many of the primary manifestations of rheumatic disease. Their targeted use in the ocular manifestations of rheumatic disease has not been as extensively studied. This paper seeks to compile the available reports on the use, efficacy, and safety of biologic agents in the treatment of ocular symptoms of rheumatic disease.

## 2. Adamantiades-Behçet's Disease

Ocular symptoms occur in 43–72% of Adamantiades-Behçet's disease (ABD) patients and affect males more commonly than females. The classic appearance is that of an anterior uveitis with a sterile hypopyon; however, presentations more often feature a posterior or diffuse uveitis with accompanying retinal vasculitis [1].

ABD is an aggressive, sight-threatening disease that requires immunosuppressive therapy to avoid vision loss. TNF-*α* antagonists are the preferred first line agent for treatment of ABD. They have proven successful in controlling symptoms, reducing ocular relapses, and significantly decreasing the daily dose of corticosteroids [2, 3]. Infliximab has emerged as the foremost agent with several prospective studies demonstrating remission of anterior and posterior segment inflammation, resolution of macular edema, and successful control of uveitis refractory to previous therapy [4, 5]. Among the anti-TNF-*α* agents, infliximab has been shown to achieve the best control of ocular signs and symptoms [1, 6].

While not as extensively studied, several reports have suggested etanercept and adalimumab are effective in controlling ABD disease severity [7, 8]. Treatment with etanercept achieves a greater response in ocular manifestations of ABD over other complications such as oral ulcers, arthritis, and skin lesions [9]. Adalimumab may induce and maintain sustained remission of refractory ocular inflammation in about 90% of patients [10].

Among the other biologic agents, rituximab has shown success in retinal vasculitis associated with ABD [11]. A case report describing the effective management of ABD with anakinra did not address ocular symptoms [12]. The ability of therapy targeting interleukin activity to reduce disease burden suggests IL-1*β* is a mediator of inflammation in ABD and may effectively treat its ocular manifestations.

## 3. Rheumatoid Arthritis

Rheumatoid arthritis (RA) gives rise to significant eye disease in 15–30% of affected patients. Characteristic presentations include keratoconjunctivitis sicca (KCS), stromal keratitis, sclerosing keratitis, scleritis, and episcleritis. KCS is by far the most common ocular manifestation (11.6%), followed by episcleritis and scleritis [13].

Dry eyes can be significantly disabling and difficult to treat. Aggressive lubrication, punctal plugs, autologous serum drops, prednisolone drops, and topical cyclosporine make up the ophthalmologist's armamentarium. This localized approach satisfactorily controls most patients; however, systemic steroids and increased methotrexate are occasionally necessary. Although not sight threatening, symptoms can exert an increasing burden as the disease progresses or increases in severity. KCS patients have various degrees of health-related quality of life impairment [14]. Although unconventional, initiation of infliximab in otherwise quiescent RA has successfully controlled KCS symptoms [15]. Evidence suggests that the indirect costs of KCS actually may outweigh the expense of biologic treatment [16].

Reinforcing the importance of TNF-*α* in the control of corneal inflammation, the TNF-*α* antagonists, infliximab, adalimumab, and etanercept have been shown to be effective therapies for RA-associated keratitis. They have shown differing levels of efficacy [17–19]. Infliximab has been shown to be the most effective agent to control RA-associated keratitis [20, 21]. Rituximab has been used successfully in the treatment of severe peripheral ulcerative keratitis (PUK) demonstrating prior resistance to anti-TNF agents [22]. However, a case of bilateral PUK following treatment with rituximab has been reported. Causation was not established [23].

Necrotizing scleritis is the most destructive form of scleritis and has considerable ocular morbidity. In patients with RA, it is associated with a high mortality, especially when not treated with immunosuppressants. Anti-TNF-*α* agents have the most evidence supporting their use and efficacy in scleritis. Certolizumab pegol has been shown to control scleritis in a patient with RA who had failed other TNF-*α* antagonists [24].

Inflammation control in the eyes of RA patients remains a challenge. Head to head comparison trials of the many biologics have not been completed. A large review of several individual studies indicated that TNF-*α* antagonists (certolizumab, adalimumab, infliximab, etanercept), the B-cell inhibitor (rituximab), and the IL-6 blocker (tocilizumab) are superior to T-cell costimulation inhibitor (abatacept) and the IL-1 blocker (anakinra). However, none of the comparisons between these biologics reached statistical significance [25].

## 4. Juvenile Idiopathic Arthritis

Uveitis occurs in 10%–15% of patients with juvenile idiopathic arthritis (JIA) and represents the primary cause of uveitis in childhood. Contrary to the red painful eye seen in adults with uveitis; the inflammation seen in children is often asymptomatic and bilateral, with an indolent chronic course. Severe vision loss, even blindness, occurs in an unacceptably high percentage of patients with one-quarter of children with JIA becoming blind in one eye [26].

Topical steroids are the first line of therapy, but only around 40% of patients respond to such treatment. The associated risks of increased intraocular pressure and cataract are particularly unappealing in a pediatric population. An investigation into the effectiveness of the three most popular anti-TNF-*α* agents, etanercept, infliximab, and adalimumab, found infliximab to be more effective than etanercept. No statistically significant conclusion was drawn regarding adalimumab [27]. Infliximab, however, was found to have a high rate of side effects in a prospective study [28]. The preferred anti-TNF-*α* agent is adalimumab, which is more effective against uveitis than etanercept and better tolerated by children [29].

Use of biologic agents not targeting TNF-*α* has only recently been published. Rituximab has reported efficacy in patients refractory to treatment with TNF-*α* antagonists [30]. Preliminary studies of high-dose intravenous administration of IL-2 antagonist, daclizumab demonstrate a reduction in active inflammation in JIA-associated anterior uveitis [31]. In a prospective study of abatacept in cases refractory to anti-TNF-*α* treatment, all seven children exhibited decreased anterior segment inflammation. However, only one demonstrated complete resolution with this treatment [32]. A head-to-head comparison of these newer agents has not been performed.

## 5. Sjogren's Syndrome

KCS, or severe dry eye, is the hallmark of Sjogren's syndrome (SS) and indicates an autoimmune attack on the lacrimal gland. Therapeutic options for the debilitating xerophthalmia are currently limited to symptomatic relief with aggressive artificial lubrication, autologous serum eye drops, topical cyclosporine, topical corticosteroids, and punctal occlusion.

Understanding the inflammatory cascade involved in SS suggests elevated levels of proinflammatory cytokines (e.g., IL-1*α*, IL-1*β*, IL-6, TNF*α*) and immunoactivators (e.g., ICAM-1, CD40, CD40 ligand) play a role the ocular symptoms [33]. Newer treatment strategies are targeting these pathways as therapeutic options. Preliminary studies of the anti-TNF-*α* agents were promising; however, a large randomized trial failed to demonstrate a difference in response between placebo and an infliximab-treated group [34]. Similarly, etanercept was also no more effective than placebo in a 12-week study [35].

While a recent double-blind, randomized, placebo-controlled trial indicated that rituximab is effective and safe in the treatment of patients with SS, ocular signs and symptoms were not among the measures demonstrating improvement with treatment [36]. A case report did report improvement in subjective and objective measures of xerophthalmia in 2 patients treated with rituximab [37].

## 6. Seronegative Spondyloarthropathy

The seronegative spondyloarthropathies, ankylosing spondylitis (AS), psoriatic arthritis (PsA), inflammatory bowel disease (IBD), and reactive arthritis (ReA) represent a group of diseases that share clinical, genetic, and pathological characteristics. They share an association with HLA-B27, an absence of positive rheumatoid factor (negative serostatus), and extra-articular features, such as involvement of eyes, skin, and genitourinary tract.

Anterior uveitis reportedly occurs in up to 30% of patients with AS. There is now accumulating evidence that targeted anti-TNF-*α* therapy is highly effective in spondyloarthritis [38]. Patients taking anti-TNF-*α* agents exhibited significantly reduced rates of recurrence of anterior uveitis in the major trials, with stronger protection afforded by infliximab and adalimumab [39, 40]. Other biologics have not shown as much promise; abatacept failed to show improvement in any outcome measures in one prospective study [41].

The classic triad of ReA includes arthritis, nongonococcal urethritis, and conjunctivitis. However, ocular manifestations may also include acute anterior uveitis. Current opinion is that because TNF-*α* drives the pathogenesis of reactive arthritis and suggests TNF-*α* antagonists will be efficacious therapeutic tools. Like AS, definitive studies are lacking but isolated case reports support the use of anti-TNF-*α* agents [42, 43].

Biologic treatment of the uveitis associated with PsA, like the other seronegative spondyloarthropathies, centers on the anti-TNF-*α* therapies [44]. Evidence suggests infliximab provides the greatest response among the anti-TNF-*α* agents [45, 46].

Inflammatory bowel disease (IBD), like the other spondyloarthropathies, can be associated with an acute anterior uveitis. It also can present with a keratitis or scleritis. Successful control of the ocular inflammation has been seen in IBD patients using TNF-*α* antagonists [47]. Of the TNF-*α* inhibiting therapies, etanercept is ineffective in controlling both the systemic symptoms of IBD and the associated uveitis [48].

## 7. Relapsing Polychondritis

Fifty-nine percent of patients with relapsing polychondritis (RP) have ocular components of their disease. The most common manifestation is scleritis, seen in 41% of patients; uveitis is seen in one-quarter of patients; conjunctivitis, episcleritis, keratitis, and retinal vasculitis are seen less frequently [49]. Immunosuppressive chemotherapy is usually required to successfully treat the ocular manifestations of RP, especially nodular and necrotizing scleritis [50]. Infliximab was shown to diminish ocular manifestations [51].

## 8. Systemic Vasculitic Disease

Giant cell arteritis (GCA) can cause an incredibly rapid total or near total loss of vision. Without prompt recognition and initiation of high-dose intravenous steroids, bilateral vision loss may result in up to 50% of individuals. Maintenance therapy with biologic agents is an attractive theory, as it would allow avoidance of chronic steroids and the associated morbidity. Studies of TNF-*α* antagonists have mixed results. Infliximab was not shown to be effective in a prospective randomized trial [52]. However, a case of reported steroid-resistant GCA was treated successfully with adalimumab [53]. Rituximab was also effective in reducing inflammatory markers in a patient with GCA refractory to treatment with corticosteroids [54].

Ocular manifestations are very common in Wegener's granulomatosis (WG), affecting approximately half of patients. Scleritis, keratitis, orbital disease, and less commonly retinovasculitis or uveitis are all potential manifestations of WG [55]. The other ANCA-associated vasculitides, microscopic polyangiitis, and Churg-Strauss present much less frequently with ophthalmic complications. Reversal of vision loss was seen in a case of severe posterior scleritis in WG treated with infliximab [56]. Rituximab has emerged as an effective tool in treating ocular manifestations of WG [57, 58]. Recent evidence suggests it is equivalent to cyclophosphamide for the induction of remission, with particular efficacy at inducing remission in patients with relapsing disease [59]. It was shown to be superior to infliximab by a small prospective study [60].

## 9. Anterior Uveitis Induced by Anti-TNF Agents

As discussed above, extensive evidence supports the efficacy of TNF-*α* antagonists in the treatment of uveitis associated with rheumatologic disease. Paradoxically, use of these agents has been implicated to cause uveitis. Several anecdotal case reports suggested an association between use of these agents and development of uveitis [61]. Subsequently, a review of medication adverse event registries not only confirmed this observation but also suggested etanercept caused a greater number of reported uveitis cases compared to infliximab and adalimumab [62]. A retrospective review reported a frequency of 1 case per 100 patient-year for patients treated with a TNF-*α* antagonist for seronegative spondylopathy [63].

## 10. Conclusion

The last decade has seen a dramatic increase in the number and nature of biologic agents. We continue to expand our knowledge of rheumatologic disease and the role of the inflammatory cascade in the ocular manifestations of those diseases. Systemically, administered small molecular antibodies and antagonists have become valuable tools in the treatment of refractory ophthalmic symptoms of rheumatic disease. In certain cases, these agents can even be considered primary therapeutic options.

Recently, two humanized monoclonal antibodies targeting vascular endothelial growth factor-A (VEGF-A), ranibizumab (Lucentis) and bevacizumab (Avastin) have revolutionized the treatment of eye diseases such as age-related macular degeneration. These agents are delivered via an intraocular injection and are effective and well tolerated [64]. Preliminary safety studies to evaluate toxicity of intravitreal injection of TNF*α* inhibitors have been performed in rabbits with experimental uveitis, with promising results [65, 66]. Future treatment of ocular manifestations of rheumatic disease will certainly build upon the documented efficacy of biologic agents and the ability to locally inject these antibodies and antagonists.

## Figures and Tables

**Table 1:** Biologic agents.

Biologic agent	Trade name	Mechanism of action
TNF-*α* blockers		

Infliximab	*Remicade *	Chimeric monoclonal antibody against TNF-*α*
Etanercept	*Enbrel *	TNF receptor-IgG fusion protein
Adalimumab	*Humira *	Human monoclonal antibody against TNF-*α*
Certolizumab pegol	*Cimzia *	PEGylated Fab of a humanized TNF inhibitor monoclonal antibody
Golimumab	*Simponi *	Humanized monoclonal antibody against TNF-*α*

Lymphocyte inhibitors		

Rituximab	*Rituxan *	Chimeric monoclonal antibody against CD20
Abatacept	*Orencia *	Selective inhibitor of T-cell costimulation

Anti-interleukin antibodies		

Anakinra	*Kineret *	IL-1 receptor antagonist
Daclizumab	*Zenapax *	Humanized monoclonal antibody to IL-2 receptor
Tocilizumab	*Actemra *	Humanized monoclonal antibody to IL-6R
Basiliximab	*Simulect *	Chimeric monoclonal antibody to the CD25

Specific receptor antibodies		

Efalizumab	*Raptiva *	CD11a, a pan-leukocyte surface marker, inhibitor
Alefacept	*Amevive *	CD2 inhibitor
Alemtuzumab	*Campath-1H *	CD52, a pan-lymphocyte antigen, antagonist

Anti-VEGF-A antibodies		

Ranibizumab	*Lucentis*	Monoclonal antibody fragment (Fab) targeting VEGF-A
Bevacizumab	*Avastin *	Monoclonal antibody targeting VEGF-A
